# Impact of Early Albumin Use for Resuscitation in Patients With Septic Shock and Cirrhosis

**DOI:** 10.1155/cjgh/8637440

**Published:** 2025-05-09

**Authors:** Hannah M. Brinkman, Kianoush B. Kashani, Alice Gallo de Moraes, Kristin C. Cole, Douglas A. Simonetto, Andrea M. Nei

**Affiliations:** ^1^Department of Pharmacy, Mayo Clinic, Rochester, Minnesota, USA; ^2^Division of Pulmonary and Critical Care Medicine, Mayo Clinic, Rochester, Minnesota, USA; ^3^Division of Nephrology and Hypertension, Mayo Clinic, Rochester, Minnesota, USA; ^4^Department of Quantitative Health Sciences, Mayo Clinic, Rochester, Minnesota, USA; ^5^Division of Gastroenterology and Hepatology, Mayo Clinic, Rochester, Minnesota, USA

## Abstract

**Background:** The choice of resuscitation fluid remains debated for patients with septic shock. While patients with cirrhosis may benefit from albumin administration, the efficacy of albumin for resuscitation in cirrhotic patients with septic shock remains unclear.

**Methods:** This is a historical cohort study of patients with cirrhosis admitted for septic shock to the intensive care unit (ICU) at a tertiary referral hospital from January 2007 to May 2017. Patients were stratified based on using albumin for fluid resuscitation within six hours of ICU admission. The primary outcome was the percentage of time during the first 48 h of ICU admission that patients were alive and shock-free. Linear regression was used to compare this outcome between groups, and a multivariable analysis was performed to account for baseline differences between study populations.

**Results:** Of the 132 patients with cirrhosis admitted for septic shock, albumin was administered within the first six hours of ICU admission for 84 patients (64%). The albumin and nonalbumin groups had similar percentages of shock-free time during the first 48 h of ICU admission (9.0% vs. 20.2%, *p* = 0.073) and ICU length of stay (5.6 vs. 3.7 days, *p* = 0.093). No differences were observed in clinical outcomes of end-organ dysfunction, such as the need for kidney replacement therapy or mechanical ventilation.

**Conclusion:** Administration of albumin during the first 6 h of ICU admission as an adjunctive resuscitation fluid to crystalloids was not associated with improved shock-free time in the ICU or clinical outcomes in patients with cirrhosis and septic shock.

## 1. Introduction

Fluid therapy is essential for resuscitation during sepsis and septic shock to augment cardiac output and oxygen delivery capacity. The Surviving Sepsis Guidelines recommend intravenous (IV) crystalloid solutions as the first fluid choice, while albumin can be considered in patients requiring large fluid volumes [1]. Albumin is thought to expand and maintain the intravascular space longer than crystalloids after bolus administration. Albumin also has anti-inflammatory, antioxidant, scavenger, and endothelial stabilization properties, which are ideal characteristics in the profound inflammatory response of sepsis [2, 3]. Although several studies have shown that adjuvant albumin fluid resuscitation may improve outcomes in septic patients compared to crystalloids alone, others have not demonstrated benefits, and albumin costs significantly more than crystalloids [4–7].

One patient population that may benefit from albumin administration during septic shock is those with cirrhosis. Cirrhosis as a comorbidity has been associated with increased mortality in septic shock [8]. Patients with cirrhosis often have low albumin levels compared to the general population, reduced blood pressures at baseline, an impaired immune response, excessive oxidative stress, and are prone to fluid accumulation in the extravascular space. Albumin administration has reduced mortality in patients with cirrhosis with other disease states, such as spontaneous bacterial peritonitis and hepatorenal syndrome. Still, there is a lack of data on albumin use in cirrhosis patients with septic shock [9, 10].

Early albumin use in patients with cirrhosis and sepsis has been evaluated in two studies, which demonstrated that using albumin for initial fluid resuscitation led to higher hypotension reversal at 3 h compared to crystalloids alone [11, 12]. However, patients on IV vasopressors at enrollment were excluded. We hypothesized that using adjunctive albumin in initial fluid resuscitation for septic shock among cirrhotic individuals improves short and long-term clinical outcomes. To evaluate this hypothesis, we conducted this cohort study to assess if using albumin as an adjunctive resuscitation fluid to crystalloids in patients with cirrhosis and septic shock would improve the percentage shock-free time within the first 48 h of ICU admission.

## 2. Methods

### 2.1. Study Design and Patient Population

**Figure 1:**
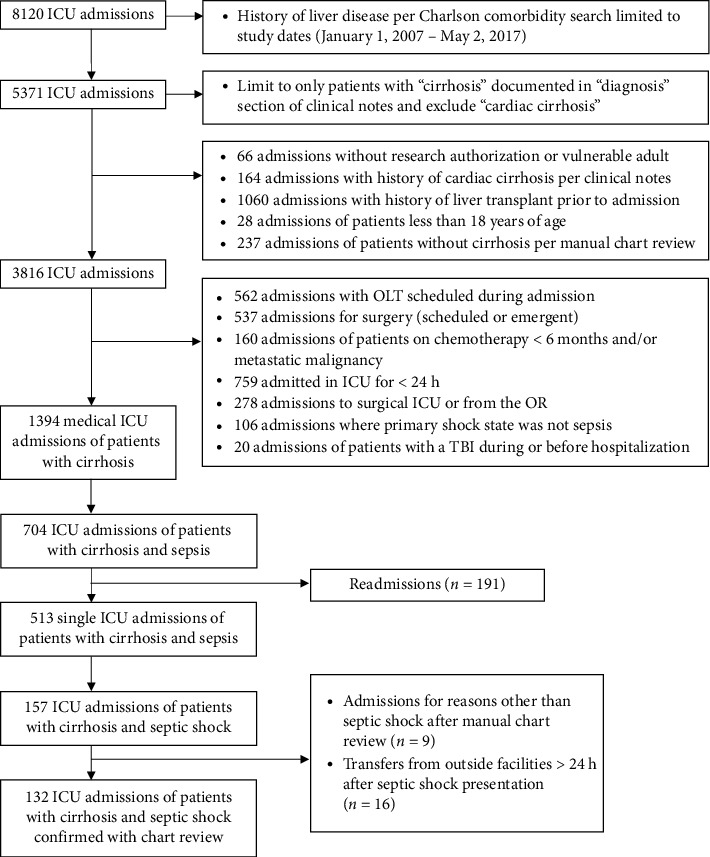
The flowchart of patient selection and allocation. Abbreviations: ICU, intensive care unit; OLT, orthotopic liver transplantation; OR, operating room; TBI, traumatic brain injury.

Patients ≥ 18 years old with cirrhosis and septic shock admitted to an ICU at Mayo Clinic Hospital-Rochester between January 1, 2007, and May 2, 2017, were included in this 10-year observational cohort study. The Mayo Clinic Institution Review Board approved this study protocol (#21-009069) and waived informed consent for patients with Minnesota Research Authorization. This study utilized data points from a previously validated dataset of septic shock patients meeting Sepsis-3 criteria for septic shock. Patients with a history of cirrhosis or liver disease admitted to the ICU were identified from the ICU Datamart using Charlson Comorbidity and advanced language processing searches related to past medical history inclusive of “cirrhosis or “liver disease” [13]. After this initial search, their liver disease was confirmed with manual chart review of liver biopsy or other supporting imaging. We excluded patients who had a history of cardiac cirrhosis, received chemotherapy within 6 months or had metastatic cancer, had undergone liver transplantation before or during their ICU admission, underwent emergent surgical intervention within the 24 h before admission, stayed in the ICU less than 24 h, admitted for a primary shock state aside from sepsis, had a traumatic brain injury before or during hospitalization, were readmissions, transferred from another inpatient admission, or were vulnerable populations including prisoners and pregnant patients (Figure 1).

### 2.2. Definitions and Outcomes

Septic shock was defined as a sequential organ failure assessment (SOFA) score ≥ 2, administration of at least 1 L of IV fluid and an IV antibiotic within 24 h of ICU admission time, and the need for vasopressors during the first 6 h after ICU admission [14]. A manual chart review of ICU admission notes confirmed the diagnosis of septic shock. Shock reversal time was defined as being independent of all IV vasopressors for at least 24 h. Shock-free time was defined as patients being alive and being independent of all IV vasopressors. Percentage shock-free time was determined as the proportion of time during the first 48 h after ICU admission that patients were alive and shock-free.

Septic shock treatment at our institution is centered on timely antibiotic administration, prompt fluid resuscitation, and norepinephrine utilization as the first-line vasopressor in accordance with sepsis guidelines [1]. The engaged ICUs in this study have incorporated a sepsis resuscitation bundle following the sepsis guidelines, with the initial fluid bolus being crystalloid [15]. Patients could receive albumin as part of fluid resuscitation at the discretion of the managing clinician. Patients in this study were stratified based on the use of 5% or 25% albumin administration in the first 6 h of ICU admission. Aside from the total length of hospitalization and mortality within 28 days, patients were observed for their ICU length of stay. The multidisciplinary critical care teams determined treatment for all patients, which was not impacted by this observational study.

The primary outcome was the percentage of time during the first 48 h of ICU admission that patients were alive and shock-free. Secondary outcomes included clinical markers of end-organ function, such as a change in SOFA score during the first 48 h of ICU admission, ICU-acquired acute kidney injury (AKI), need for kidney replacement therapy (KRT) or mechanical ventilation, ICU length of stay and mortality, and 28-day all-cause mortality.

### 2.3. Data Collection

The demographic, laboratory, and clinical outcomes variables were abstracted from electronic health records [13]. Data were either previously validated or manually reviewed after random selection of 10% of the sample [16]. The start and discontinuation times of vasopressors, amount of albumin administration in the first 6 h, admission diagnosis of septic shock, the presumed site of infection, and etiology of cirrhosis were confirmed with manual chart review for all included patients.

The acute physiology and chronic health evaluation III (APACHE III) score and the Charlson comorbidity index (CCI) were calculated at ICU admission, and SOFA scores were documented daily. The model for end-stage liver disease (MELD) scores were calculated using laboratory values during hospitalization that were closest to ICU admission. For MELD and MELD-Na calculations, patients with values outside the set upper and lower limit ranges were adjusted by considering the serum creatinine (SCr) lower limit as 1 mg/dL and the upper limit as 4 mg/dL (if dialysis twice in the last week, creatinine set to 4 mg/dL), bilirubin lower limit as 1 mg/dL, international normalized ratio (INR) lower limit as 1, sodium lower limit as 125 mmol/L and the upper limit as 137 mmol/L, and the lower limits were inputted for patients with missing values, with the exception of sodium where the upper limit of 137 mmol/L was used [17]. The MELD-Na was only calculated for those with a MELD score > 11. Vasopressor use was reported as the cumulative norepinephrine base equivalent dose during ICU admission and the average dose during the time of administration [18, 19].

Patients who had not undergone KRT in the previous 6 months were assessed for a new requirement of continuous KRT or intermittent hemodialysis. Those who had an increase in SCr by 0.3 mg/dL within 48 h or > 1.5 times their baseline SCr within the first 7 days were determined to have an ICU-acquired AKI [20]. Baseline serum creatinine was the closest SCr up to 90 days prior to ICU admission [21].

### 2.4. Data Analysis

Continuous variables were described using means with standard deviations or medians with interquartile ranges and compared between groups with an independent Student's *t*-test or Wilcoxon-Rank-Sum test per data distribution. Categorical variables were described using frequencies and percentages and analyzed with chi-square or Fisher's exact test if the number of observations in either group was less than five, as appropriate. Linear regression was used to assess the association between the albumin group and the percentage of time during the first 48 h of ICU admission that patients were alive and shock-free. Logistic regression was used to assess the outcome of death. Time-to-event adverse effects were compared between groups using Cox proportional hazard regression and rates calculated using the Aalen-Johansen method, with death treated as a competing risk for any outcomes besides death. Clinical outcomes were also analyzed by multivariable regression models to account for clinically relevant variables, including early or no early albumin administration, MELD, SOFA score, baseline hemoglobin, and antibiotics before admission. All analyses were performed using SAS version 9.4 (SAS Institute, Inc. Cary, NC), and *p* values < 0.05 were statistically significant.

## 3. Results

### 3.1. Patients

Of the 3816 ICU admissions with cirrhosis between January 1, 2007, and May 2, 2017, a manual chart review identified 132 patients who met all eligibility criteria (Figure 1). Albumin was administered within the first six hours of ICU admission for 84 patients (64%) (albumin group), and 48 patients (36%) received only crystalloids during this timeframe for resuscitation (nonalbumin group). The baseline demographics were similar between groups. The most common etiology of cirrhosis was alcohol (31%), followed by nonalcoholic fatty liver disease (27%) and chronic hepatitis C infection (11%). In patients with a baseline albumin value (*n* = 84), 25 (29.8%) had an albumin of < 2.5 g/dL, 23 (27.4%) had an albumin of 2.5–3 g/dL, and 36 (42.9%) had an albumin > 3 g/dL. In the albumin group, 58 patients received only 5% albumin (69%), 12 patients received 25% albumin (14%), and 14 patients (17%) received both albumin concentrations. The median amount of albumin administered in the first 6 h was 0.54 g/kg (IQR 0.32, 0.89) (Table 1) with 17 patients (20.5%) receiving > 1 g/kg in the first 6 h.

The mean SOFA score on the day of ICU admission was higher in the albumin group compared to the nonalbumin group (12.6 vs. 10.7, *p* = 0.003), as was the median MELD-Na score (27.9 vs. 21.4, *p* < 0.001). However, the mean APACHE III score on day 0 of ICU admission was similar between the two groups (60.9 vs. 61.6, *p* = 0.79). The presumed site of infection varied, with more patients in the albumin group admitted for an abdominal source (48% vs. 27%) and fewer for a genitourinary infection (2% vs. 15%). Norepinephrine was the most common vasopressor infusion administered during the first 6 hours of ICU admission (Table 1). Numerically more patients in the albumin group received 2 or more vasopressors compared to the no albumin group 29/84 (34.5%) vs 10/48 (20.8%) (*p* = 0.097).

### 3.2. Outcomes

The albumin and nonalbumin groups had similar percentages of shock-free ICU time during the first 48 h (9.0% vs. 20.2%, *p* = 0.073) and ICU length of stay (5.6 vs. 3.7 days, *p* = 0.093). Other analyses of shock, including shock-free time and the composite outcome of shock-free time survival during the first 48 h of ICU admission, were not different between groups. No differences were observed in clinical outcomes of end-organ dysfunction, such as the need for KRT or mechanical ventilation and change in SOFA score from ICU admission (day 0) to day 1. Mortality during hospitalization (39% vs. 10%, *p* = 0.030) and within 28 days of ICU admission (60% vs. 40%, *p* = 0.046) were significantly higher in the albumin group, but mortality during ICU stay was similar (29% vs. 17%, *p* = 0.12) (Table 2). The total vasopressor exposure ICU stay in norepinephrine equivalents was significantly higher in the albumin group (42.0 vs. 15.3 mg, *p* = 0.008). The total volume administered and fluid balance in the first 6 h were similar between groups (Table 3).

Upon multivariable analysis, albumin was not shown to impact any clinical outcomes, including the percentage of shock-free time in the first 48 h, mortality, or length of stay (Tables 4, 5 and 6). Higher SOFA scores and age and female sex were associated with a lower percent shock-free time in the first 48 h (Table 4). A higher SOFA score was associated with an increased risk of death during ICU stay (OR 1.33, 95% CI 1.16–1.53, *p* < 0.001) and within 28 days of ICU admission (OR 1.19, 95% CI 1.04–1.36, *p* = 0.011), but a lower risk of death during hospitalization (OR 0.73, 95% CI 0.64–0.85, *p* < 0.001) (Table 5). Similarly, a higher SOFA score was associated with less likelihood of both ICU and hospital discharge and a longer length of stay (Table 6).

## 4. Discussion

### 4.1. Summary of Key Findings

The early use of albumin as an adjunctive resuscitation fluid to crystalloids in patients with cirrhosis and septic shock was not associated with increased shock-free time or its percentage during ICU admission. Mortality during ICU admission was not different, but there was a significant increase in hospital and 28-day mortality in those who received albumin. The patients in the albumin group may have had greater severity of illness at baseline, reflected by the significantly higher vasopressor requirements, MELD-Na, and SOFA score, which may have led to a higher proportion of patients with refractory shock receiving albumin. Significantly more patients in the albumin group had abdominal infectious sources, which are associated with a higher inflammatory response compared to other categorized sources of infection [22]. The increase in patients with a presumed abdominal infectious source receiving albumin may have been due to concern for spontaneous bacterial peritonitis and the previously described mortality benefit of albumin administration in this subset of patients. Despite these factors, there was no difference in mortality observed during ICU admission and percent of shock-free ICU time, which would be more attributable to the intervention within 6 h of ICU admission.

### 4.2. Relationship With Previous Studies

Prior studies have demonstrated a 20% increase in ICU and hospital mortality in patients with cirrhosis and sepsis compared to those without cirrhosis, with a reported mortality of 60%–100% for septic shock [23]. In this study, the mortality rates in both groups were lower, possibly due to the exclusions of patients with active malignancy, cardiac cirrhosis, or liver transplantation during ICU admission. Mortality could also be lower in our center since there is a specific ICU for patients with liver-related critical illnesses and a dedicated gastroenterology service.

While the Surviving Sepsis Guidelines suggest administering albumin in addition to crystalloids for high-volume resuscitation, few studies have examined the impact of this combination for fluid resuscitation or the outcomes of early albumin administration in sepsis [1]. Two studies in the general population evaluated using albumin and crystalloids within the first 6 h of sepsis resuscitation and did not find improvements in survival, hemodynamics, or vasopressor utilization [24, 25]. Our study adds similar results to the literature on patients with cirrhosis, which were not well represented in previous studies.

One randomized prospective study in patients with cirrhosis presenting with sepsis-induced hypotension and not yet on vasopressors found that 20% albumin administration (0.5–1 g/kg over 3 h) was superior to PlasmaLyte (30 mL/kg over 3 h) in achieving a target MAP > 65 at 3 h (62% vs. 22%, *p* < 0.001) [12]. However, this effect was not sustained at 24 or 48 h. The 28-day mortality rate was similar between groups, and 22% of patients in the albumin group discontinued the treatment due to adverse effects (primarily pulmonary edema or bronchospasm) compared to no patients in the PlasmaLyte group. Our study assessed albumin in combination with crystalloid fluid compared to crystalloid alone, which may be a more applicable approach in early fluid resuscitation, given the urgency to improve hemodynamics and restore end-organ perfusion.

The open-label, randomized trial by Philips and colleagues reported that 5% albumin for initial fluid resuscitation in patients with septic cirrhosis had more hypotension reversal at 3 h per vital sign improvements than crystalloids, and a greater proportion of patients surviving at 7 days in the albumin group [11]. However, they did not report clinical markers of end-organ dysfunction or outcomes beyond 7 days and excluded patients with septic shock, unlike our primary study population. Conversely, the increased probability of survival was not found in three other studies examining the impact of albumin in patients with cirrhosis presenting with infections aside from spontaneous bacterial peritonitis. Notably, these studies either used noncontemporary sepsis definitions for inclusion criteria or excluded patients with suspected sepsis or septic shock [26–28].

Other studies evaluating albumin use longitudinally in the ICU have identified possible subgroups that may benefit from albumin administration. A large cohort study including patients with sepsis and cirrhosis (25% with septic shock) found that despite a higher mortality in patients who received albumin, after adjustment for time-dependent administration and baseline confounders, albumin was associated with reduced 28-day mortality [29]. Notably, this study suggested a potential window of benefit with albumin administration including serum albumin of 2.5–3 g/dL, lactate > 2 mmol/L, MAP less than 60 mmHg, or vasopressor norepinephrine equivalent between 0.2–0.3 mcg/kg/min. When comparing albumin dosing strata, albumin doses ≤ 1 g/kg/day were associated with reduced mortality, while doses above this threshold were not. Safety concerns of pulmonary complications with excessive albumin doses have been suggested in other studies [11, 28]. These studies do not focus on the initial resuscitation period in sepsis or septic shock but may inform future analyses of sufficient power to discern the patient subtype which may benefit most from albumin administration.

### 4.3. Implication of Study Findings

Cirrhosis is an independent risk factor for mortality in sepsis, which may be attributed to baseline low systemic vascular resistance, subclinical cardiomyopathy, central hypovolemia, accumulation of fluid in the extravascular space, and impaired immunologic function [30]. Fluid resuscitation in this patient population is challenging with the increased blood volume in the splanchnic vasculature relative to central hypovolemia at baseline, along with the potential for albumin to increase the splanchnic circulation more than the central compartment, as albumin gradually redistributes outside central circulation [31]. Albumin is proposed to increase intravascular colloid osmotic pressure longer than crystalloids and is thought to have anti-inflammatory, antioxidant, and endothelial stabilization properties that could reduce end-organ dysfunction [2, 3]. However, these outcomes were not demonstrated in our study, in which both groups had a similar incidence of KRT initiation, need for supported ventilation, and change in SOFA score from ICU day 0 to day 1. Given the important baseline differences between groups in our study despite statistical adjustments and inability to fully account for these differences in our outcomes analyses, our findings should be considered hypothesis-generating. Further clinical investigation would be needed to connect the pathophysiological advantages of albumin use in patients with cirrhosis and septic shock to clinically impactful outcomes. While the theoretical benefits of using albumin in cirrhosis patients who present with septic shock may be attractive to clinicians, multidisciplinary groups have yet to recommend albumin over balanced crystalloids for infections outside of spontaneous bacterial peritonitis, and our findings support this rationale [32–34].

### 4.4. Strengths and Limitations

Strengths of this study include enrolling patients with albumin exposure early during resuscitation and excluding surgical patients who may have a fluid or vasopressor requirement unrelated to septic shock. While the sepsis guidelines suggest that 30 mL/kg of IV crystalloid be given in early resuscitation, our study sepsis definition required 1 L of crystalloid within 24 h of ICU admission time, given the lack of precise data supporting the routine use of 30 mL/kg of fluids in this patient population [11, 35, 36]. The inclusion of invasive organ system supportive treatments, such as KRT and mechanical ventilation, in the outcomes provides further insight into the morbidities and perfusion derangements associated with septic shock rather than hemodynamic parameters and mortality alone.

There are several limitations to this study. First, the observational design limited the ability to control the scenarios for which albumin was administered, along with the data that were available for analysis. Second, the accuracy of the data was dependent on availability and charting, which could lead to misclassification bias; however, the study population of interest was manually validated for both cirrhosis and septic shock without reliance on diagnostic codes for patient selection. Furthermore, the outcomes and measurements of interest are routinely assessed during the treatment of septic shock patients and pulled directly from the electronic health record, reducing the potential for data gaps or inaccuracies.

Third, baseline laboratory data was limited to 24 h before ICU admission, and baseline SCr was reported as the closest SCr up to 90 days before admission. Fourth, all baseline data was restricted to that available at our institution, and these values may not represent the actual organ function of the patient before septic shock onset. Nonetheless, utilization of the data available prior to admission may represent real-world decision-making from the managing clinician regarding fluid administration within the first few hours of admission for septic shock. Fifth, given the small sample size of our study, it is possible that it was underpowered to detect a difference in clinical outcomes. However, given the lack of data in this population, our observations contribute to the study question that warrants further investigation by larger studies. Sixth, the period of our study with the changes in sepsis management and prescribing practices that occurred over time is another limitation, but contemporary definitions of septic shock were utilized for all patients. Seventh, the use of albumin as a primary resuscitation fluid instead of an adjunct and the impact of the amount of albumin on clinical outcomes were not evaluated due to the limited sample size but could be considered for future studies. Finally, while we attempted to adjust for confounders in multivariable analysis, we were limited by outcome frequency and could not adjust for unknown confounders in the clinical outcomes.

## 5. Conclusion

In patients with cirrhosis and septic shock, using albumin during the first 6 h of ICU admission as an adjunctive resuscitation fluid to crystalloids was not associated with improving shock-free time, end-organ function, length of stay, or mortality. There was an increase in hospital mortality associated with albumin administration, which may have been reflective of the albumin group having a higher severity of illness, yet ICU mortality was not different. Fluid resuscitation in patients with cirrhosis and hypovolemia in the central compartment from sepsis is a balancing act, and the lack of positive findings from our study concerning albumin use provides further guidance for clinicians seeking to optimize outcomes in this complex patient population. While our results were not significant and trended away from a benefit of albumin use, we recognize that this may have been due to the size of our population in congruence with variations in baseline characteristics. Although ideally an interventional study is conducted, we hope in the interim our study can provide information to clinicians and, moreover, perhaps inspire them to conduct such studies and further investigate this important topic.

## Figures and Tables

**Table 1:** Demographics and baseline characteristics.

Variables	Early albumin (*n* = 84)	No early albumin (*n* = 48)	*p* value
Age, years mean (SD)	60.9 (9.5)	64.0 (12.4)	0.14
Sex			0.27
Male	52 (62%)	25 (52%)	
Female	32 (38%)	23 (48%)	
Race			0.39
White	76 (91%)	45 (94%)	
American Indian/Alaskan native	3 (4%)	0 (0%)	
Black or African American	0 (0%)	1 (2%)	
Other	4 (5%)	2 (4%)	
Unknown	1 (1%)	0 (0%)	
Weight, kg, median (IQR)	85.9 (24.5)	88.3 (29.5)	0.62
BMI, kg/m^2^, median (IQR)	27.6 (23.3, 34.9)	29.9 (23.8, 38.5)	0.14
Etiology of cirrhosis			0.60
Alcohol	28 (33%)	13 (27%)	
NAFLD	19 (23%)	16 (33%)	
Hepatitis C	10 (12%)	5 (10%)	
Other	27 (32%)	14 (29%)	
APACHE III score, mean (SD)	60.9 (23.7)	61.6 (23.3)	0.79
SOFA score, day 0, mean (SD)	12.6 (3.6)	10.7 (3.5)	0.003
MELD score, median (IQR)	26.2 (21.1, 31.9)	19.7 (12.8, 24.5)	< 0.001
MELD-Na score, median (IQR)	27.9 (23.6, 32.9)	21.4 (12.8, 26.8)	< 0.001
Comorbidities			
Charlson comorbidity index			
Median (IQR)	8 (6, 11)	9 (6, 12)	0.53
CHF	29 (35%)	18 (38%)	0.73
CKD	43 (51%)	21 (44%)	0.41
Dialysis	14 (17%)	6 (13%)	0.52
Diabetes	32 (38%)	24 (50%)	0.18
Presumed site of infection			0.007
Bloodstream	17 (20%)	13 (27%)	
Abdominal	40 (48%)	13 (27%)	
Genitourinary	2 (2%)	7 (15%)	
Respiratory	20 (24%)	10 (21%)	
SSTI/Osteomyelitis	5 (6%)	2 (4%)	
Other	0 (0%)	3 (6%)	
Baseline lab values (ICU admission), median (IQR)			
Albumin (*n* = 84) (mg/dL)	3.1 (2.4, 3.5)	2.5 (2.2, 2.9)	0.052
Creatinine (*n* = 132) (mg/dL)	2.1 (1.1, 3.6)	1.8 (1.1, 3.9)	0.65
Lactate (*n* = 122) (mmol/L)	2.7 (1.7, 4.4)	2.4 (1.6, 3.4)	0.39
INR (*n* = 114)	2.0 (1.7, 2.2)	1.7 (1.3, 2.1)	0.017
Direct bilirubin (*n* = 92) (mg/dL)	2.5 (1.4, 7.4)	1.1 (0.6, 1.9)	0.005
Hemoglobin (*n* = 132) (g/dL)	8.6 (7.7, 9.8)	10.2 (9.0, 11.7)	< 0.001
Home medications			
Beta-blockers	23 (27%)	11 (23%)	0.57
Diuretics	56 (67%)	26 (54%)	0.15
Antibiotics	46 (55%)	15 (31%)	0.009
Lactulose	45 (54%)	14 (29%)	0.007
Corticosteroids	7 (8%)	8 (17%)	0.15
Albumin amount within 6 h of ICU admission, g, median (IQR)			
Total	50 (25, 75)	—	—
5%	37.5 (25, 50)		
25%	0 (0, 25)		
Grams per patient kg	0.54 (0.32, 0.89)		
Vasopressor infusions within 6 h of ICU admission (could be multiple)			
Norepinephrine	71 (85%)	40 (83%)	0.86
Vasopressin	30 (36%)	12 (25%)	0.20
Dopamine	10 (12%)	10 (21%)	0.17
Epinephrine	4 (5%)	0 (0%)	0.12
Phenylephrine	8 (10%)	2 (4%)	0.26

*Note:* Numbers indicate as *N* (%) or median (IQR), where applicable, APACHE III, acute physiology and chronic health evaluation III score.

Abbreviations: BMI, body mass index; CHF, congestive heart failure; CKD, chronic kidney disease; INR, international normalized ratio; MELD score, model for end-stage liver disease score; MELD-Na score, model for end-stage liver disease-sodium score; NAFLD, nonalcoholic fatty liver disease; SD, standard deviation; SOFA score, sequential organ failure assessment score; SSTI, skin, and soft tissue infection.

**Table 2:** The effect of albumin on outcomes in patients admitted with septic shock.

	Early albumin (*n* = 84)	No early albumin (*n* = 48)	*p* value
Shock duration, median (IQR)			
Percent shock free and alive time in first 48 h	9.0 (3.8, 29.1)	20.2 (4.8, 56.9)	0.073
Shock free time in the first 48 h (hours)	4.3 (1.8, 14.0)	9.7 (2.3, 27.3)	0.073
Shock free and alive time in first 48 h (hours)	4.3 (1.8, 14.0)	9.7 (2.3, 27.3)	0.073
Shock free time in ICU (days)	1.2 (0.5, 3.0)	1.1 (0.6, 2.6)	0.88
Percent of shock-free ICU time	35.6 (16.1, 60.0)	41.0 (27.2, 67.4)	0.20
Duration of vasopressor (days)	2.3 (1.3, 4.4)	1.5 (0.9, 3.2)	0.021
Length of stay, days, median (IQR)^a^			
ICU LOS	5.6 (2.8, 9.3)	3.7 (1.9, 5.6)	0.093
ICU admission to hospital discharge	12.7 (7.4, 19.8)	8.3 (5.3, 14.5)	0.005
Overall hospital LOS	16.3 (8.5, 25.6)	8.6 (5.4, 17.1)	0.003
Mortality			
Death during ICU stay	24 (29%)	8 (17%)	0.12
Death during hospitalization	33 (39%)	10 (21%)	0.030
Death within 28 days of ICU admission	40/67 (60%)	15/38 (40%)	0.046
Kidney outcomes			
New KRT during ICU stay (*n* = 127)	16/70 (23%)	7/42 (17%)	0.43
ICU-acquired AKI	28 (33%)	11 (23%)	0.21
Pulmonary outcomes			
Need for any ventilation support	53 (63%)	36 (63%)	0.16
Invasive ventilation^b^	46 (56%)	30 (64%)	0.15
Invasive ventilator support duration (days)	3.8 (2.3, 6.3)	1.6 (1.0, 4.1)	0.44
Any ventilator support duration (days)	3.2 (1.3, 6.2)	1.6 (0.6, 4.2)	0.12
Proportion of any ventilation support free ICU time	69.9% (23.8%, 100.0%)	59.4% (32.1%, 99.7%)	0.54
End organ function outcomes			
SOFA score change from ICU admission (day 0) to day 1, mean (SD)	−2.8 (3.5)	−3.3 (3.3)	0.27

*Note:* Numbers indicate as *N* (%) or median (IQR), where applicable.

Abbreviations: AKI, acute kidney injury; KRT, kidney replacement therapy; LOS, length of stay; SOFA score, sequential organ failure assessment score.

^a^Survival methods were used to compare these outcomes between groups, where discharge alive was considered the event, and patients who died were censored at death.

^b^Survival methods were used to compare these outcomes between groups, where patients were followed from ICU admission to invasive ventilation (event), death (competing risk), or ICU discharge (censor), whichever came first.

^c^Survival methods were used to compare these outcomes between groups, where patients were followed from the start to the end of invasive ventilation (event) or death (censor).

**Table 3:** Fluid and other medication administration.

	Early albumin (*n* = 84)	No early albumin (*n* = 48)	*p* value
Total volume of fluid in first 6 h (mL per kg)	44.1 (20.6, 73.9)	38.1 (13.8, 62.4)	0.25
Fluid balance 1^st^ 6 h ICU (mL)	3001.1 (1440.1, 5240.9)	3585.7 (912.2, 4596.2)	0.55
Total vasopressor dose during ICU stay (NE equivalent) (mcg/kg)	42.0 (12.7, 91.8)	15.3 (6.2, 42.4)	0.008
Average vasopressor dose (NE equivalent) (mcg/kg/min)	0.011 (0.006, 0.019)	0.007 (0.004, 0.013)	0.040
Hydrocortisone IV use during ICU stay	50 (60%)	25 (52%)	0.41
Midodrine use during ICU stay	31 (37%)	10 (21%)	0.055

*Note:* Numbers indicate as *N* (%) or median (IQR), where applicable.

Abbreviation: NE, norepinephrine.

**Table 4:** Multivariable model for percent of first 48 h ICU time alive and shock-free.

	% of ICU time that was shock-free	
	Estimate (95% CI)	*p* value
Albumin (early vs. Not early)	−8.4 (−18.4 to 1.6)	0.10
Age (per year)	−0.6 (−1.0 to −0.2)	0.008
Sex (F vs. M)	−11.9 (−20.7 to −3.2)	0.008
SOFA, ICU day 0	−3.0 (−4.3 to −1.7)	<0.001
MELD	0.0 (−0.6 to 0.6)	0.96
Charlson comorbidity index	0.0 (−1.2 to 1.3)	0.97
Baseline hemoglobin	1.6 (−1.1 to 4.2)	0.24
Home medications—antibiotics (Y vs. N)	5.7 (−3.6 to 14.9)	0.23

Abbreviations: MELD score, model for end-stage liver disease score; SOFA score, sequential organ failure assessment score.

**Table 5:** Multivariable models for mortality.

	Death during ICU stay		Death during hospitalization		Death within 28 days of ICU admission	
	Odds ratio (95% CI)	*p* value	Odds ratio (95% CI)	*p* value	Odds ratio (95% CI)	*p* value
Albumin (early vs. not early)	0.97 (0.34–2.73)	0.95	0.90 (0.33–2.44)	0.83	0.77 (0.29–2.03)	0.60
Age (per year)			0.95 (0.91–0.99)	0.015	1.08 (1.03–1.13)	< 0.001
SOFA, ICU day 0	1.33 (1.16–1.53)	< 0.001	0.73 (0.64–0.85)	< 0.001	1.19 (1.04–1.36)	0.011
MELD					1.05 (1.01–1.13)	0.033
Baseline hemoglobin	0.76 (0.57–1.02)	0.070	1.60 (1.18–2.16)	0.003	0.61 (0.45–0.81)	< 0.001

Abbreviations: MELD score, model for end-stage liver disease score; SOFA score, sequential organ failure assessment score.

**Table 6:** Multivariable model for length of stay.

	ICU LOS		Overall hospital LOS	
	Hazard ratio (95% CI)	*p* value	Hazard ratio (95% CI)	*p* value
Albumin (early vs. Not early)	0.93 (0.56–1.55)	0.78	0.72 (0.43–1.23)	0.23
Age (per year)	1.01 (0.99–1.03)	0.46	1.04 (0.67–1.63)	0.86
Sex (F vs. M)	0.90 (0.59–1.39)	0.64	0.98 (0.95–1.00)	0.092
SOFA, ICU day 0	0.82 (0.76–0.88)	< 0.001	0.91 (0.85–0.99)	0.018
MELD	1.01 (0.98–1.04)	0.67	0.99 (0.96–1.02)	0.40
Charlson comorbidity index	0.97 (0.91–1.03)	0.34	1.04 (0.98–1.11)	0.19
Baseline hemoglobin	1.05 (0.93–1.19)	0.41	1.15 (1.00–1.33)	0.047
Home medications—antibiotics (Y vs. N)	1.24 (0.79–1.95)	0.35	1.28 (0.78–2.11)	0.33

*Note:* Hazard ratios > 1 mean more likely to be discharged/shorter LOS. These take deaths into account.

Abbreviations: MELD score, model for end-stage liver disease score; SOFA score, sequential organ failure assessment score.

## Data Availability

All data are presented in this article. The archived medical charts of each case included in this study cannot be made available for consultation by others who are not a part of the research team to protect confidentiality.
